# Bibliographical

**Published:** 1856-10

**Authors:** 


### EDITORIAL DEPARTMENT.

### BIBLIOGRAPHICAL.

*The Microscope: its History, Construction and Application.* By Jabez Hogg, Esq., M. R. C. S., &c. Ingram & Co., London.

The use of the microscope, until within the last few years, was confined to men of means and of scientific research; the books that have been published upon the subject have either been too expensive or too scientific for our intelligent artisans—not so with the volume before us, comprising as it does, in a popular and cheap form, a cyclopœdia of information on all subjects relating to the microscope. In the days of Sir John Hill the microscope was not only a philosophic toy, but was most imperfect in its construction, and its application was also limited to the discovery of huge monsters in drops of water, things depicted upon a disc as large as a household warming pan. Mr. Hogg not only illustrates vegetable life, but every thing relating to animal fungoid diseases, human skin, &c. with upwards of three hundred wood cuts.

The microscope has revealed to us that many of the skin diseases attacking the human frame are but other forms of the same growth of parasitic fungi, or cryptogamia, another low form of plant, presenting at first filaments simple, then ramified, and formed by a single elongated cell, or several cells placed end to end, as in those of the yeast-plant. The disease known as *ringworm* infesting the heads of children, is one out of forty-eight different species of *cryptogamia.* The conditions of growth of this low form of vegetable life on the human body are the same as in other situations. Dr. Gudden, who has lately published a work upon *cutaneous Diseases caused by "Parasitic Growths,"* describes ringworm under the name of *porrigo-fungus ;* the spores of which are round on the upper, and filamentous on the under surface. Whenever the healthy chemical processes of nutrition are impaired, and the incessant changes between the solids and the fluids slacken, then the skin may furnish a proper soil for the fungi to take root in, should the sporules come in contact with it. That dreadful disease known as cancer will no doubt ultimately prove of vegetable growth, or a conversion of the nutritive animal cell into that of a fungoid vegetable cell.

The Rev. S. G. Osborne, during the cholera visitation of 1854, endeavored to direct public attention to the very general distribution of fungi He says : "Only those who have closely studied those fungi can be aware how very minute and yet how systematically formed they are. Preparations of a dozen different species, taken from the grape, potato, parsnip, bean, cucumber, cineraria, veronica, &c., many of which have been in fluid for more than a year, retain their form as perfectly as if only taken from the plant a day. No two are alike in form, but all are alike in this—under the very high powers of the microscope they show an external hyaline case, with a second utricle, or inner case, full of minute spores.

If a few leaves of the infected haulm of the potato are taken, and gently shaken over a piece of black paper, a quantity of very fine white powder is obtained; place a little of this in fluid, under a power of 500 linear; every atom of this powder will resolve itself into a distinct cell, somewhat of the form of an ace of spades, varying more or less in size, from about 3-5000ths of an inch in length. There will be seen a well defined outline of an inner cell, in which are many hundred greenish-looking spores; some of the cells will burst, and by using a still higher power it will be seen that these have all the shapes and characteristics of the parent cell. Several of them lie easily between the lines on a micrometer, which lines are just 1-5000th of an inch apart.

There can scarcely be one spot of earth on which these fungi do not fall in their thousands. Insoluble in nature, they wait where they fall, the growth of the particular plant for which each has its own affinity, that if that plant grows on that spot, its enemy is near, on the very soil from which it is to draw life. But I further believe that there must be some peculiar disposition yet to be developed in the plant before the fungus will act upon it, to its own rapid development, and the destruction of the said plant.

Our limited knowledge of the matter does not forbid the supposition that there may be some, even among the purely vegetable fungi, which might in certain conditions of the human body, when taken into the frame, produce immediate severe constitutional disturbance. The *sarcina* may be cited as an instance of this fact. It strikes us, however, as far more probable, that from drains and cesspools—reservoirs as they are for excrementitious animal matter—may emanate certain specific fungi, the spores of which, under certain conditions of atmosphere, would be given out in such quantities, and in such minute particles, as easily to be carried about by every current of air. Persons in health may inhale and swallow these spores, and escape injury from them. Other persons, depressed physically, from local or accidental causes, may afford to them just the *pabulum* which will develop their poisonous quality.

Many animal organisms, such as infusorial animalcules and their ova, are frequently found floating about in the air, as well as the fungi spoken of.

Animals, birds, insects and fishes alike suffer from the ravages of fungi. One of the most prevalent of these, observed among our domestic pets, is the fungi growing over the upper surface of the gold-fish; death is almost certain when this white fungoid disease once commences its ravages. Great devastation is at times committed amongst silkworms by the ***botrytis***, causing a disease called ***muscardine***, just as they are about to enter the chrysalis state.

The author deserves the thanks of all lovers of science for the vast amount of industry and scientific research he has shown in the collection of his materials, &c., and the simple and forcible manner in which he treats the various subjects, directing the student in popular phraseology how to investigate and practice the use of the microscope. R.

---

***Dental Anæsthesia.—Painless Tooth Extraction by Congelation.*** By Richard Quinton.

We have received from the author a copy of this work and have read it with much interest. Our views of this *questio vexata* is well expressed in the preface. "In the enthusiasm engendered by new discoveries, a tendency to exaggeration is often manifested." How far the writer has been successful in his efforts to "steer clear of this, by demonstrating the general and particular physiological effects of the agent in question; and while commending its virtue, avoiding the error of ascribing to it omnipotence," we shall not say.

The treatise contains much matter that will well repay a careful perusal; it also contains information, good enough in its place, but quite irrelevant to the subject in hand. This occasional irrelevance, a certain high flown style at times out of place, with here and there symptoms of mere bookmaking craft, are blemishes in an otherwise excellent little work. We should have been better pleased had the author given an illustrated description of his apparatus for the *gradual* application and withdrawal of cold. Rapid freezing, with no measures taken to arrest too sudden reaction, Mr. Quinton unhesitatingly condemns. Is there no advocate of this truly American method of applying Dr. Arnott's discovery prepared to bring forward the results of his experience in its defence? We stand open to conviction.

